# New Jersey State Veterinary Society

**Published:** 1891-09

**Authors:** A. T. Sellers

**Affiliations:** Secretary


*New Jersey State Veterinary Society.*—The annual meeting of the New Jersey State Veterinary Society, was held in New Brunswick, Thursday August 6, 1891, in White Hall Hotel.

The meeting was called to order at 2:30 P. M. President E. L. Loblein, occupying the chair. On roll call the following members answered to their names:

Drs. Jos. Hopkins, J. C. Corlies, J. N. Krowl, E. L. Loblein, W. H. Lowe, E. R. Mercer, L. R. Sattler, A. T. Sellers.

The minutes of the previous session were read and approved. The address of the President followed. The Board of Censors recommended the admission to membership of the following: Drs. E. R. Ogden, E. C. Batten, W. F. Harrison, and T. H. Ripley. The report was received and the doctors named were duly elected to membership.

The following applications for membership were received and referred to the Board of Censors for action: Drs. E. Landes, of Camden; W. Gray, of Newton; E. D. Bachman, Phillipsburg; J. M. Whittpan, Jersey City; J. P. Lowe, Paterson; S. C. Tremaine, Bridgeton.

The election of officers resulted as follows: President, Dr. James Hopkins; Vice-President, Dr. W. H. Lowe; Treasurer, Dr. A.H. McIntosh; Secretary, Dr. A. T. Sellers. Board of Censors: Drs. E. R. Mercer, J. N. Krowl, E. R. Ogden, E. C. Batten, and E. L. Loblein.

The newly elected President was escorted to the chair, and made an address of interest to those present. The following resolution was then adopted, which is intended to probe the supposed existance of contagious pleuro-pneumonia in and around Newark, where it is reported cattle are being condemned because of the disease, and afterward the meat sold to the public for food :

*Whereas*, The alleged existance of contagious pleuro-pneumonia among the cattle around Newark, N. J., and the action of the Bureau of Animal Industry, in quarantining and destroying herds, has induced much hardship among the small owners of cows, and

*Whereas*, Many complaints have been made to veterinarians by owners of cattle, that no such disease exists in that vicinity, therefore be it

*Resolved*, That this society shall appoint a committee of five to investigate the methods pursued by the agents of the Bureau of Animal Industry, and if deemed advisable have a special meeting called to consider the action necessary.

Drs. E. C. Batten, E. L. Loblein and L. R. Sattler, were appointed essayists for the next meeting.

Drs. E. R. Mercer, W. H. Lowe, and James Hopkins, were selected as delegates to the next Annual meeting of the United States Veterinary Medical Association.

The essayists for the meeting being unavoidably absent, several hours were consumed in discussing Colic in its different forms, and its treatment. Many interesting and valuable points were brought out, notably the Eserine treatment of flatulent colic. Congestive Colic, the newly recognized form, received lengthy attention of the greatest value to those present. The absent members missed a good meeting.

At six o'clock P. M., we adjourned to the Banquet Hall, where an elegant repast was served.

Paterson was selected as the next place of meeting.

A. T. SELLERS, *Secretary*.

